# The effect of polyphenols and vitamin E on the antioxidant status and meat quality of broiler chickens fed low-quality oil

**DOI:** 10.5194/aab-62-287-2019

**Published:** 2019-05-23

**Authors:** Magdalena Mazur-Kuśnirek, Zofia Antoszkiewicz, Krzysztof Lipiński, Joanna Kaliniewicz, Sylwia Kotlarczyk

**Affiliations:** Department of Animal Nutrition and Feed Science, University of Warmia and Mazury, Olsztyn, Poland

## Abstract

The aim of this study was to determine the effect of vitamin E and polyphenols on the antioxidant potential and meat quality of broiler chickens fed diets supplemented with low-quality oil. The experimental materials comprised 120 male Ross 308 broilers (six treatments, 10 replications, two birds per replication). Dietary supplementation with vitamin E and/or polyphenols was applied in the following experimental design: group I (negative control) – without supplementation without low-quality oil; group II (positive control) – without supplementation + low-quality oil; group III – supplementation with 100 mg kg${}^{-1}$ of vitamin E+ low-quality oil; group IV – 200 mg kg${}^{-1}$ of vitamin E + low-quality oil; group V – 100 mg kg${}^{-1}$ of vitamin E and 100 mg kg${}^{-1}$ of polyphenols + low-quality oil; group VI – 200 mg kg${}^{-1}$ of polyphenols + low-quality oil. Rapeseed oil oxidised under laboratory conditions was added to the diets of broiler chickens from groups II to VI. The applied antioxidants had no effect on the growth performance of chickens fed oxidised oil. Increased dietary inclusion levels of vitamin E and/or polyphenols improved the antioxidant status in the blood and increased the content of non-enzymatic antioxidants in the liver and breast muscles of broilers fed low-quality oil. The tested antioxidants had no influence on carcass quality parameters in chickens fed oxidised oil. However, birds fed diets with the addition of vitamin E were characterised by a higher gizzard weight and higher pH of gizzard digesta. Dietary supplementation with vitamin E and polyphenols or polyphenols alone contributed to a lighter colour and lower pH of breast muscles and an increase in the content of fat and ash in the breast muscles of broilers fed oxidised oil. The breast muscles of birds given 100 or 200 mg kg${}^{-1}$ of supplemental vitamin E were characterised by higher concentrations of n-6 polyunsaturated fatty acids (PUFAs) and hypocholesterolemic fatty acids (DFAs), a more desirable DFA/OFA ratio, and a lower atherogenicity index (AI). Polyphenols combined with vitamin E can be a valuable component of diets for broiler chickens when the problem of low-quality oil occurs.

## 1 Introduction

The quality of oil as a source of energy in poultry diets influences growth performance and the health status of flocks. Low-quality oil added to broiler chicken diets decreases productivity (Anjum et al., 2002; McGill et al., 2011), increases mortality (Anjum et al., 2004) and decreases the quality of animal products (Zhang et al., 2011). Diets rich in polyunsaturated fatty acids (PUFAs) increase susceptibility to lipid peroxidation and decrease the antioxidant capacity of animals. Rancid lipids that undergo auto-oxidation processes contain free-radical-generating substances that exert adverse effects on the health status of birds. Oxidation reactions produce harmful peroxides that are converted into hydrocarbons, ketones, alcohols, organic acids and aldehydes (Baião and Lara, 2005) including malondialdehyde (MDA) with mutagenic and carcinogenic properties. Oxidation reactions also decrease the content of vitamins A and E and carotenoids (Bayraktar et al., 2011; Koch and Hill, 2016). Increased production of reactive oxygen species (ROS) disrupts redox balance and contributes to oxidative stress with harmful implications for health (Koch and Hill, 2016).

Supplementation with antioxidants is an effective method of minimising the adverse consequences of low-quality oils in animal diets. Recent years have witnessed growing levels of awareness among consumers and food producers regarding the origin of feed additives in livestock nutrition. The demand for products of natural origin continues to increase.

Polyphenols are substances of plant origin which possess antioxidant properties and may reduce the negative effects of oxidative stress (Brenes et al., 2016; Lipiński et al., 2017). Their antioxidant potential is comparable with that of the major biological antioxidants: α-tocopherol and ascorbic acid (Surai, 2014). Polyphenols also have immunomodulatory, anti-inflammatory and bactericidal properties (Landete, 2013).

The results of in vitro and in vivo studies demonstrated that polyphenols deliver positive effects in animal nutrition (Alonso et al., 2002,; Torres et al., 2002; Viveros et al., 2011; Gessner et al., 2013; Kamboh and Zhu, 2014; Chen et al., 2018), including in animals whose diets contain oxidised oils or oils rich in PUFAs (Sobotka et al., 2012; Lu et al., 2014; Kishavy et al., 2016).

The research hypothesis postulated that polyphenols from onions and grape seeds can partially replace vitamin E in terms of antioxidant activity in broiler chickens fed oxidised oil. The aim of this study was to determine the effect of polyphenols and increased inclusion levels of vitamin E on the antioxidant potential and meat quality of broilers fed diets supplemented with low-quality oil.

## 2 Materials and methods

### 2.1 Animals and materials

The experiment was approved by the Local Ethics Committee for Animal Experimentation in Olsztyn, Poland. The experimental materials comprised 120 male Ross 308 broiler chickens (six treatments, 10 replications, two birds per replication). The chickens were kept in cages without litter (two birds per cage). The experiment lasted 35 d. The body weights of birds were determined at weekly intervals. Feed intake and mortality rates were also monitored, and the data were used to calculate the feed conversion ratio (FCR).

The birds were fed ad libitum starter (1–14 d of age) and grower (15–35 d of age) diets in mash form, which fully met the nutrient requirements of broiler chickens (Nutrient Requirements of Poultry, 2005), and they had free access to water.

The Proviox Nucleus preparation (Provimi, France) used in the study contains polyphenols obtained from onions (quercetin and flavonols) and grape seeds (catechins, flavonols, procyanidins and anthocyanidins). Broiler chicken diets were supplemented with 50 % vitamin E in the form of dl-α-tocopheryl acetate. The experimental design is shown in Table 1. Rapeseed oil oxidised under laboratory conditions was added to the diets of broiler chickens from groups II to VI: 25 g kg${}^{-1}$ (starter diets) and 40 g kg${}^{-1}$ (grower diets). Rapeseed oil was heated at a temperature of 80 ${}^{\circ }$C (Elkon CL-65 laboratory heater with forced air circulation) for 14 d and was aerated for 8 h daily (KNF Lab Laboport vacuum pump). Rapeseed oil had the following parameters: acid value (AV) – 54.0 mg KOH g${}^{-1}$; peroxide value (POV) – 55.7 meq $O_{2}$ kg${}^{-1}$; malondialdehyde (MDA) content – 67.39 mg kg${}^{-1}$.

**Table 1:** Experimental design.

Group	Additives	Low-quality oil
I	–	–
II	–	+
III	100 mg kg${}^{-1}$ of vitamin E	+
IV	200 mg kg${}^{-1}$ of vitamin E	+
V	100 mg kg${}^{-1}$ of vitamin E and 100 mg kg${}^{-1}$ of polyphenols	+
VI	200 mg kg${}^{-1}$ of polyphenols	+

### 2.2 Methods

Feed and breast meat samples were assayed for dry matter (DM), crude ash, crude protein (CP), ether extract (EE) and crude fibre (CF) by standard methods (AOAC, 2005). Vitamin E concentrations were measured by chromatography (HPLC – high-performance liquid chromatography) (Shimadzu, Japan), according to the method described by Polish Standard PN-EN ISO 6867 (2002). The amount of metabolisable energy and the content of amino acids and minerals in broiler chicken diets were calculated according to the Nutrient Requirements of Poultry (2005). The composition and energy value of diets without supplemental vitamin E are presented in Table 2.

**Table 2:** Composition (%), content of vitamin E (mg kg${}^{-1}$) and energy value (ME${}_{N}$ MJ kg${}^{-1}$) of broiler chicken diets.

Specification	Diets	
	Starter (1–14 d of age)	Grower (15–35 d of age)
Wheat	63.5	69.5
Soybean meal	30.0	23.0
Rapeseed oil	2.5	4.0
Limestone	1.5	1.0
Premix${}^{*}$	2.5	2.5
Nutrient content and energy value		
Energy ME${}_{N}$	12.34	13.14
Crude protein	22.60	20.00
Crude fibre	2.79	2.25
Crude fat	4.20	5.49
Lys	1.31	1.15
Met	0.57	0.54
Met + Cys	0.96	0.89
Thr	0.85	0.74
Trp	0.27	0.23
Ca	1.00	0.80
Pp	0.42	0.40
Na	0.18	0.18
Vitamin E	10.50	10.30

${}^{*}$ Premix composition: 480 000 IU of vitamin A, 160 000 IU of vitamin D${}_{3}$, 120 mg of vitamin K${}_{3}$, 120 mg of vitamin B${}_{1}$, 280 mg of vitamin B${}_{2}$, 1800 mg of niacin (B${}_{3}$), 500 mg of pantothenic acid, 200 mg of vitamin B${}_{6}$, 1000 µg of vitamin B${}_{12}$, 8000 µg of biotin, 28000 mg of choline chloride, 60 mg of folic acid, 2000 mg of Fe, 4000 mg of Mn, 600 mg of Cu, 4000 mg of Zn, 36 mg of I, 12 mg of Se, 2800 mg of salinomycin, 100 000 U E.-1.4 B-xyl (Danisco), 24 000 FTU (unit of phytase activity) of 6-phytase (Phyzyme Xp), 52 g of Na, 131 g of Ca, 73 g of total P, 120 g of available P, 100 g of lysine, 100 g of methionine, 30 g of threonine.

Blood samples were collected from broiler chickens on day 35. The activity of superoxide dismutase (SOD) and glutathione peroxidase (GSH-Px) was determined in heparinised whole blood. Total antioxidant status (TAS) and the concentrations of vitamins A and E were determined in the blood serum. Blood samples were stored at a temperature of -20 ${}^{\circ }$C until analysis.

Total antioxidant status, SOD and GSH-Px activity were determined with the use of the Randox kit according to the manufacturer's recommendations. The activity of SOD was measured as the percent inhibition of the rate of formazan dye formation (505 nm wavelength). The activity of GSH-Px in heparinised whole blood was determined with the use of cumene hydroperoxide. In the presence of glutathione reductase and NADPH (nicotinamide adenine dinucleotide phosphate), oxidised glutathione is converted to a reduced form, and NADPH is oxidised to NADP+ (340 nm wavelength). The total antioxidant status was determined in the blood serum based on the colour reaction with the ABTS (2,2'-azinobis-3-ethylbenzothiazoline-6-sulfonic acid) stock solution and metmyoglobin. The antioxidants present in the analysed samples reduce the intensity of bluish-green colour proportionally to their concentrations (600 nm wavelength). The analysis was performed using a spectrophotometer (EPOLL-30 ECO, Poll Ltd., Warsaw, Poland).

Serum samples (1 mL) were deproteinised with anhydrous ethanol (1 mL), extracted with 5 mL of n-hexane (Vortex 5') and centrifuged (3000 rpm, 10 min, 4 ${}^{\circ }$C). The resulting supernatant in the amount of 4 mL was evaporated to dryness under nitrogen, diluted in 1 mL of 96 % ethanol. Tocopherol and retinol concentrations were measured by chromatography (HPLC) (Shimadzu, Japan), according to the method described by Rettenmaier and Schüep (1992).

Vitamin E equivalent (vitamin EEq) was calculated based on the activity of tocopherols relative to α-T. If α-T=1, then β-T=0.4, γ-T=0.1. δ-T=0.01; vitamin EEq $=C_{1}+0.4C_{2}+0.1C_{3}+0.01C_{4}$, where $C_{1}$ is α-T content (mg kg${}^{-1}$ DM), $C_{2}$ is β-T content (mg kg${}^{-1}$ DM), $C_{3}$ is γ-T content (mg kg${}^{-1}$ DM, $C_{4}$ is δ-T content (mg kg${}^{-1}$ DM); 1 IU of vitamin E =0.67 mg of vitamin EEq (Eittenmiller et al., 1998).

At 35 d of age, 10 birds per group were slaughtered and carcass quality was evaluated. Samples of fresh breast muscles and liver were assayed for the concentrations of lipophilic vitamins (retinol, total tocopherols) as described by Rettenmaier and Schüep (1992), hydrophilic vitamins (vitamin C) as described by Omaye et al. (1979), and thiobarbituric acid-reactive substance (TBARS) values according to Sørensen and Jørgensen (1996).

The percentage content of breast muscles in the carcass and abdominal fat, heart and liver in live body weight was determined. Samples of fresh breast muscles were assayed for proximate chemical composition (AOAC, 2005) and meat quality parameters including acidity (pH15 and pH24, with the WTW 3310 pH meter and a Hamilton Double Pore probe), colour in the CIE $L^{*}a^{*}b^{*}$ system ($L^{*}$ – lightness; $a^{*}$ – redness; $b^{*}$ – yellowness, CIE, 1978; with the Hunter Lab Mini Scan XE Plus spectrophotometer), 24 h post mortem natural drip loss (as described by Honikel, 1998), and water-holding capacity (by the method developed by Grau and Hamm, 1952, and modified by Pohja and Niinivaara, 1957).

Breast muscle samples were assayed for the concentrations of fatty acids after methylation of extracted lipids (Peisker, 1964) by chromatography, including saturated fatty acids (SFAs), monounsaturated fatty acids (MUFAs), n-3 and n-6 polyunsaturated fatty acids (PUFAs), hypercholesterolemic fatty acids (OFAs) and hypocholesterolemic fatty acids (DFAs), in accordance with the criteria proposed by Barowicz et al. (2000) and Leskanich and Noble (1997). The fatty acid percentages were calculated with the Microsoft Excel (Microsoft Excel 2013) software and the mean value of each fatty acid was used to calculate the following indexes:

- peroxidisability index (PI) – (% monoenoic ×0.025) + (% dienoic ×1) + (% trienoic ×2) + (% tetraenoic ×4) + (% pentaenoic ×6) + (% hexaenoic ×8) (Arakawa and Sagai, 1986);
- atherogenicity index (AI) – (C12:0+4×C14:0+C16:0) / [(ΣMUFA +ΣSPUFA (n-6) and (n-3)] (Ulbricht and Southgate, 1991);
- thrombogenic index (TI) – (C14:0+C16:0+C18:0)/[(0.5 ×ΣMUFA +0.5×ΣSPUFA (n-6) +3×ΣSPUFA (n-3) + (n-3)/(n-6)] (Ulbricht and Southgate, 1991).

Segments of the gastrointestinal tract were emptied to determine their weights and pH of their contents. Dressing percentage was calculated as the ratio of carcass weight to live weight.

**Table 3:** Growth performance of broiler chickens fed low-quality oil.

Specification	Group							
	I negative	II positive	III vit. E	IV vit. E	V vit. E	VI polyphenols	SEM	P
	control	control	100 mg kg${}^{-1}$	200 mg kg${}^{-1}$	100 mg kg${}^{-1}+$	200 mg kg${}^{-1}$		
					polyphenols 100 mg kg${}^{-1}$			
n	10	10	10	10	10	10	–	–
Body weight (g)								
– Day 7	118.15	114.45	118.75	127.40	124.05	127.25	2.03	0.342
– Day 21	717.50	605.00	711.50	717.30	762.30	712.45	16.46	0.161
– Day 35	2055.95	1899.56	2134.60	2035.44	2176.80	2010.30	36.35	0.353
Body weight gain (g)								
– Day 7	81.05	77.45	83.45	89.82	84.85	89.68	1.79	0.391
– Day 21	680.40	568.78	676.20	679.83	723.10	674.40	16.12	0.262
– Day 35	2018.80	1861.88	2099.30	1997.73	2137.60	1972.25	36.30	0.314
Feed intake (g)								
– Day 7	77.10	67.78	73.23	78.13	73.63	77.54	1.56	0.394
– Day 21	904.15	780.89	904.03	895.45	951.58	919.66	21.23	0.352
– Day 35	3125.78	2952.05	3171.58	3043.69	3202.58	3115.78	70.16	0.341
FCR (kg kg${}^{-1}$)								
– Day 7	0.95	0.88	0.88	0.87	0.87	0.89	0.01	0.192
– Day 21	1.33	1.36	1.38	1.39	1.32	1.36	0.02	0.973
– Day 35	1.55	1.59	1.52	1.52	1.50	1.58	0.02	0.621
Mortality, n	1	1	0	1	0	2	–	–

The AV and POV of rapeseed oil were determined according to Polish Standards PN-88/C-04288/06 and PN-88/C-04288/10, and MDA concentration was determined as described by Esterbauer et al. (1991).

### 2.3 Statistical analysis

The results were processed statistically by one-way analysis of variance and Duncan's test. The arithmetic mean ($\bar{x}$), standard error of the mean (SEM) and the level of significance (p<0.05 and p<0.01) were given for all results. All calculations were performed in the Statistica 10 program.

## 3 Results

### 3.1 Growth performance

The applied antioxidants did not improve the growth performance of broiler chickens fed diets containing oxidised rapeseed oil (Table 3). Broiler mortality was not recorded in groups receiving diets supplemented with 100 mg kg${}^{-1}$ of vitamin E (group III) and vitamin E combined with polyphenols (group V). Birds whose diets were supplemented with antioxidants had higher final body weights and body weight gains, but the observed differences were not statistically significant.

### 3.2 Antioxidant status

At 35 d of age, no differences in TAS or SOD activity were found between groups (Table 4). The addition of polyphenols to diets (groups V and VI) increased GSH-Px activity in the blood of broiler chickens (p≤0.01) compared with birds fed diets without antioxidants containing low-quality oil (group II) and diets supplemented with vitamin E (groups III and IV).

**Table 4:** Antioxidant status of broiler chickens fed low-quality oil.

Specification	Group							
	I negative	II positive	III vit. E	IV vit. E	V vit. E	VI polyphenols	SEM	P
	control	control	100 mg kg${}^{-1}$	200 mg kg${}^{-1}$	100 mg kg${}^{-1}+$	200 mg kg${}^{-1}$		
					polyphenols 100 mg kg${}^{-1}$			
n	10	10	10	10	10	10	–	–
Blood								
TAS (mmol L${}^{-1}$)	1.21	0.91	1.14	1.06	1.04	1.07	0.03	0.112
SOD (U mL${}^{-1}$)	53.28	43.57	51.60	50.50	48.49	46.49	1.40	0.489
GSH-Px (U mL${}^{-1}$)	24.91${}^{A}$	20.96${}^{\mathrm{Bb}}$	21.96${}^{B}$	22.10${}^{B}$	25.25${}^{A}$	23.45${}^{a}$	0.34	<0.001
Retinol (µg g${}^{-1}$)	0.75${}^{B}$	0.75${}^{B}$	0.71${}^{B}$	0.81${}^{B}$	0.70${}^{B}$	1.09${}^{A}$	0.02	<0.001
Total tocopherols (µg g${}^{-1}$)	2.25${}^{\mathrm{DE}}$	1.91${}^{E}$	7.25${}^{C}$	13.14${}^{A}$	9.87${}^{B}$	2.84${}^{D}$	0.56	<0.001
Vitamin EEq	2.02${}^{D}$	1.68${}^{\mathrm{Db}}$	7.11${}^{C}$	12.99${}^{A}$	9.71${}^{B}$	2.52${}^{\mathrm{Da}}$	0.57	<0.001
Liver								
Vitamin C (µg g${}^{-1}$)	201.75${}^{A}$	173.56${}^{\mathrm{Bb}}$	204.06${}^{A}$	194.53${}^{a}$	191.06${}^{a}$	188.56${}^{a}$	2.41	<0.001
Retinol (µg g${}^{-1}$)	66.48${}^{A}$	24.63${}^{E}$	32.92${}^{D}$	22.41${}^{E}$	42.44${}^{C}$	58.67${}^{B}$	2.36	<0.001
Total tocopherols (µg g${}^{-1}$)	5.74${}^{D}$	1.81${}^{E}$	10.09${}^{C}$	13.84${}^{B}$	17.47${}^{A}$	4.18${}^{D}$	0.75	<0.001
Vitamin EEq	3.05${}^{D}$	1.55${}^{E}$	9.88${}^{C}$	13.74${}^{B}$	15.09${}^{A}$	2.09${}^{E}$	0.74	<0.001
TBARS (mg kg${}^{-1}$)	1.01${}^{B}$	1.54${}^{\mathrm{Aa}}$	0.72${}^{\mathrm{Bc}}$	0.60${}^{\mathrm{Bc}}$	1.15${}^{\mathrm{ABb}}$	1.33${}^{\mathrm{ABa}}$	0.06	<0.001
Breast muscles								
Vitamin C (µg g${}^{-1}$)	110.51	106.99	108.35	107.12	109.40	102.94	1.33	0.373
Retinol (µg g${}^{-1}$)	0.16${}^{A}$	0.12${}^{B}$	0.17${}^{A}$	0.16${}^{A}$	0.17${}^{A}$	0.13${}^{B}$	0.01	<0.001
Total tocopherols (µg g${}^{-1}$)	2.24${}^{C}$	1.94${}^{C}$	7.61${}^{\mathrm{Bb}}$	13.85${}^{A}$	8.67${}^{\mathrm{Ba}}$	2.19${}^{C}$	0.68	<0.001
Vitamin EEq	1.87${}^{D}$	1.76${}^{D}$	7.37${}^{C}$	13.59${}^{A}$	8.51${}^{B}$	1.96${}^{D}$	0.65	<0.001
TBARS (mg kg${}^{-1}$)	0.35${}^{\mathrm{AB}}$	0.75${}^{\mathrm{Aa}}$	0.31${}^{\mathrm{bc}}$	0.07${}^{\mathrm{Bbc}}$	0.13${}^{\mathrm{Bc}}$	0.59${}^{\mathrm{ab}}$	0.06	<0.001

a, b – p≤0.05. A, B – p≤0.01.

The addition of polyphenols to diets (group VI) led to an increase (by 46 % on average, p≤0.01) in serum retinol levels in comparison with the remaining groups. Dietary supplementation with vitamin E and polyphenols increased the concentrations of total tocopherols and vitamin EEq (p≤0.01) in the blood serum of broilers compared with birds that did not receive the antioxidants.

Broiler chickens fed diets supplemented with antioxidants were characterised by higher (p≤0.01 and p≤0.05) vitamin C concentrations in the liver relative to birds that were exposed to the dietary stressor but did not receive the antioxidants (Table 4). Diets with low-quality oil contributed to a decrease in retinol levels in the liver of chickens. The addition of polyphenols and vitamin E to diets resulted in the highest concentrations of tocopherols and vitamin EEq in the liver of broilers (group V) (p≤0.01) relative to the remaining groups. Supplemental vitamin E (groups III and IV) led to a two-fold decrease in TBARS values in the liver of chickens compared with birds fed diets with rancid oil and without antioxidants (p≤0.01).

The vitamin C content of breast muscles was similar in all chickens (Table 4). The lowest retinol content (24 % on average, p≤0.01) was noted in the breast muscles of broilers fed diets with oxidised oil and without antioxidants (group II), and diets with polyphenols (group VI). Supplemental vitamin E contributed to higher concentrations of total tocopherols (10 µg kg${}^{-1}$ on average) and vitamin EEq (9.8 µg kg${}^{-1}$ on average) (p≤0.01 and p≤0.05) in the breast muscles of chickens from groups III, IV and V compared with the remaining groups. TBARS values were several-fold higher (p≤0.05 and p≤0.01) in the breast muscles of chickens fed diets with low-quality oil and without antioxidant supplementation (group II) than in the breast muscles of birds receiving vitamin E (groups III, IV and V).

### 3.3 Carcass quality

Low-quality dietary oil had no significant effect on carcass dressing percentage or the percentage content of breast muscles in the carcass (Table 5). However, dressing percentage tended to increase in broilers fed diets without rancid oil (group I) and diets containing rancid oil and supplemented with polyphenols (group VI).

**Table 5:** The effect of antioxidants on carcass quality, structure of the gastrointestinal tract and digesta pH in broiler chickens fed low-quality oil. BW stands for body weight.

Specification	Group							
	I negative	II positive	III vit. E	IV vit. E	V vit. E	VI polyphenols	SEM	P
	control	control	100 mg kg${}^{-1}$	200 mg kg${}^{-1}$	100 mg kg${}^{-1}+$	200 mg kg${}^{-1}$		
					polyphenols 100 mg kg${}^{-1}$			
n	10	10	10	10	10	10	–	–
Carcass dressing percentage (%)	70.53${}^{x}$	69.73${}^{\mathrm{xy}}$	68.19${}^{y}$	68.65${}^{\mathrm{xy}}$	69.85${}^{\mathrm{xy}}$	70.52${}^{x}$	0.26	0.064
Breast muscle content of carcass (%)	28.96	29.03	28.89	28.85	28.10	27.77	0.18	0.302
Heart content of BW (%)	0.65	0.60	0.61	0.65	0.61	0.64	0.01	0.111
Liver content of BW (%)	2.28	2.25	2.03	2.28	1.98	2.12	0.04	0.102
Abdominal fat content of BW (%)	0.32	0.36	0.37	0.35	0.34	0.31	0.02	0.966
Crop								
– Weight (g kg${}^{-1}$ BW)	4.06	4.88	4.67	4.07	4.14	4.06	0.05	0.155
– Digesta pH	4.22	4.53	4.22	4.54	4.53	4.59	0.06	0.132
Proventriculus								
– Weight (g kg${}^{-1}$ BW)	3.61	3.36	3.53	3.33	3.77	3.37	0.06	0.284
– Digesta pH	2.70	2.13	3.01	3.07	3.21	3.22	0.16	0.341
Gizzard								
– Weight (g kg${}^{-1}$ BW)	10.23${}^{B}$	12.02${}^{\mathrm{AB}}$	13.45${}^{\mathrm{Aa}}$	13.37${}^{A}$	12.10${}^{\mathrm{AB}}$	11.35${}^{b}$	0.01	<0.001
– Digesta pH	1.94${}^{B}$	2.83${}^{A}$	1.79${}^{B}$	1.86${}^{B}$	2.30${}^{\mathrm{AB}}$	2.23${}^{\mathrm{AB}}$	0.09	<0.001
Small intestine								
– Length (cm kg${}^{-1}$ BW)	80.26	82.21	75.18	78.96	77.13	74.82	0.97	0.133
– Weight (g kg${}^{-1}$ BW)	18.02	19.48	19.42	19.61	18.17	18.62	0.24	0.162
– Digesta pH	5.91	5.70	5.78	5.95	6.02	5.97	0.04	0.152
Caeca								
– Length (cm kg${}^{-1}$ BW)	18.89	18.47	18.94	19.06	18.81	18.68	0.25	0.914
– Weight (g kg${}^{-1}$ BW)	3.31	3.18	3.49	3.32	3.11	3.45	0.07	0.338
– Digesta pH	5.85	5.75	6.08	6.13	5.92	5.94	0.06	0.421

a, b – p≤0.05. A, B – p≤0.01. x, y – p≥0.05 – p≤0.1.

No differences in the percentage content of the heart, liver and abdominal fat were found between groups (Table 5). The analysed antioxidants had no significant effect on the weight and pH of the crop and proventriculus in all groups. Higher gizzard weight (by 24 % on average, p≤0.01) and higher pH of gizzard digesta (p≤0.01) were noted in chickens fed diets supplemented with vitamin E (groups III and IV). The tested antioxidants had no influence on the weight, the length and pH of small intestinal and caecal digesta.

### 3.4 Meat quality

The meat of broilers fed diets without oxidised oil (group I) and diets supplemented with 100 mg kg${}^{-1}$ of vitamin E (group III) had lower pH${}_{15}$ values (p≤0.05 and p≤0.01) than the meat of birds from the remaining groups (Table 6). The lowest value of pH${}_{24}$ (p≤0.01) was noted in the tissues of chickens receiving polyphenols (groups V and VI), and the highest value of pH${}_{24}$ was observed in birds fed diets with the highest dose of vitamin E (group IV) (p≤0.05 and p≤0.01).

**Table 6:** Physicochemical properties of breast muscles in broiler chickens fed low-quality oil. WHC stands for water-holding capacity.

Specification	Group							
	I negative	II positive	III vit. E	IV vit. E	V vit. E	VI polyphenols	SEM	P
	control	control	100 mg kg${}^{-1}$	200 mg kg${}^{-1}$	100 mg kg${}^{-1}+$	200 mg kg${}^{-1}$		
					polyphenols 100 mg kg${}^{-1}$			
n	10	10	10	10	10	10	–	–
pH${}_{15}$	6.14${}^{B}$	6.50${}^{\mathrm{Aa}}$	6.28${}^{b}$	6.37${}^{A}$	6.45${}^{A}$	6.37${}^{A}$	0.03	<0.001
pH${}_{24}$	5.96${}^{\mathrm{AB}}$	5.90${}^{\mathrm{AB}}$	5.89${}^{\mathrm{AB}}$	6.03${}^{\mathrm{Aa}}$	5.80${}^{B}$	5.87${}^{b}$	0.02	0.040
Colour								
$L^{*}$	60.59${}^{\mathrm{Bb}}$	60.47${}^{\mathrm{Bb}}$	61.08${}^{B}$	60.36${}^{\mathrm{Bb}}$	62.68${}^{a}$	63.60${}^{A}$	0.31	<0.001
$a^{*}$	5.64	5.54	5.19	5.06	5.37	5.19	0.13	0.774
$b^{*}$	15.11${}^{b}$	15.49${}^{\mathrm{ab}}$	16.53${}^{a}$	16.32${}^{a}$	16.21${}^{\mathrm{ab}}$	15.50${}^{\mathrm{ab}}$	0.16	0.042
Natural drip loss (%)	2.78	2.79	2.61	2.88	2.55	2.71	0.20	0.553
WHC (cm${}^{2}$)	5.02	4.02	4.36	4.16	4.57	4.47	0.11	0.101
Dry matter (%)	25.79	25.72	26.21	25.77	25.71	26.02	0.11	0.784
Crude protein (%)	22.32	21.99	22.79	22.43	22.03	22.55	0.14	0.615
Crude fat (%)	1.11${}^{\mathrm{Bb}}$	2.00${}^{A}$	1.58${}^{\mathrm{AB}}$	1.44${}^{\mathrm{AB}}$	1.60${}^{\mathrm{AB}}$	1.71${}^{a}$	0.08	0.047
Crude ash (%)	1.12${}^{\mathrm{Bb}}$	1.21${}^{a}$	1.25${}^{A}$	1.20${}^{a}$	1.21${}^{a}$	1.23${}^{A}$	0.01	0.013

a, b – p≤0.05. A, B – p≤0.01.

The breast muscles of broilers fed diets supplemented with polyphenols were lighter ($L^{*}$) in colour (groups V and VI) (p≤0.05 and p≤0.01) than the breast muscles of the remaining birds (Table 6). The muscle tissue of chickens fed diets supplemented with vitamin E (groups III and IV) had a higher (p≤0.05) contribution of yellowness ($b^{*}$).

No significant differences in natural drip loss or the water-holding capacity of meat were found between groups (Table 6). The analysed meat samples did not differ in the content of dry matter or protein (Table 6). Crude fat content was nearly two-fold lower in the breast muscles of broilers fed diets without oxidised oil (group I), compared with birds fed diets with oxidised oil and without antioxidants (group II) and diets with the highest inclusion level of polyphenols (group VI) (p≤0.01 and p≤0.05). The breast muscles of broiler chickens fed oxidised oil had a higher crude ash content (p≤0.05 and p≤0.01).

### 3.5 Fatty acid profile of breast muscles

The analysed breast muscles of broiler chickens did not differ in the content of SFAs (Table 7). Broilers whose diets did not contain oxidised oil (group I) were characterised by lower concentrations of total MUFAs and higher content of PUFAs, relative to the remaining groups.

**Table 7:** Percentage content of fatty acids in the breast muscles of broiler chickens.

Specification	Group						SEM	P
	I negative	II positive	III vit. E	IV vit. E	V vit. E	VI polyphenols		
	control	control	100 mg kg${}^{-1}$	200 mg kg${}^{-1}$	100 mg kg${}^{-1}$+	200 mg kg${}^{-1}$		
					polyphenols 100 mg kg${}^{-1}$			
n	10	10	10	10	10	10	–	–
SFAs	27.63	28.56	26.88	27.44	27.87	29.23	1.00	0.102
MUFAs	46.07${}^{\mathrm{Bb}}$	50.33${}^{A}$	49.24${}^{A}$	48.24${}^{a}$	48.88${}^{A}$	50.11${}^{A}$	1.99	<0.001
PUFAs	26.30${}^{\mathrm{Aa}}$	21.11${}^{\mathrm{Cc}}$	23.88${}^{\mathrm{ABb}}$	24.32${}^{\mathrm{ABb}}$	23.25${}^{\mathrm{Bab}}$	21.66${}^{\mathrm{Bc}}$	2.18	<0.001
n-3	3.82${}^{x}$	3.42	3.70	3.62	3.67	3.32${}^{y}$	0.31	0.093
n-6	20.52${}^{\mathrm{Aa}}$	16.77${}^{\mathrm{Cd}}$	18.95${}^{\mathrm{ABbd}}$	19.27${}^{\mathrm{ABbd}}$	18.48${}^{b}$	17.29${}^{\mathrm{BCc}}$	1.64	<0.001
n-6/n-3	5.41	4.91	5.13	5.33	5.07	5.21	0.34	0.211
DFAs	80.28${}^{A}$	78.12${}^{\mathrm{Bc}}$	79.87${}^{\mathrm{Aa}}$	80.01${}^{\mathrm{Aa}}$	79.26${}^{\mathrm{ab}}$	78.64${}^{\mathrm{Bb}}$	1.07	<0.001
OFAs	19.71${}^{B}$	21.88${}^{\mathrm{Aa}}$	20.13${}^{\mathrm{BCb}}$	19.99${}^{\mathrm{BCb}}$	20.74${}^{b}$	21.36${}^{a}$	1.07	<0.001
DFA/OFA	4.08${}^{A}$	3.58${}^{C}$	3.97${}^{\mathrm{ABa}}$	4.00${}^{\mathrm{ABa}}$	3.83${}^{\mathrm{ABC}}$	3.69${}^{\mathrm{BCb}}$	0.25	<0.001
AI	0.28${}^{\mathrm{Bb}}$	0.32${}^{\mathrm{Aa}}$	0.29${}^{\mathrm{Bb}}$	0.29${}^{\mathrm{Bb}}$	0.30${}^{\mathrm{bc}}$	0.31${}^{\mathrm{ac}}$	0.01	0.005
TI	0.59	0.69	0.57	0.59	0.60	0.62	0.01	0.109
PI	29.70${}^{\mathrm{Aa}}$	15.49${}^{B}$	18.40${}^{B}$	20.43${}^{b}$	18.97${}^{B}$	16.90${}^{B}$	1.26	0.008

a, b – p≤0.05. A, B – P≤0.01. x, y – p≥0.05p≤0.1. SFAs – saturated fatty acids; MUFAs – monounsaturated fatty acids; PUFAs – polyunsaturated fatty acids; DFAs – neutral and hypocholesterolemic fatty acids (UFAs – unsaturated fatty acids – + C${}_{18:0}$); OFAs – hypercholesterolemic fatty acids (C${}_{14:0}$ + C${}_{16:0})$; AI – atherogenicity index; TI – thrombogenicity index; PI – peroxidisability index.

The content of n-3 PUFAs tended to decrease in broilers whose diets were supplemented with polyphenols only relative to the negative control group (group I). The breast muscles of birds given polyphenols only (group VI) and broilers fed low-quality oil without antioxidants were characterised by a lower content of n-6 PUFAs, DFAs, a lower DFA/OFA ratio and a higher content of OFAs, compared with the remaining groups. PI percentage in the breast muscle of chickens fed diets without oxidised oil was higher than in the remaining birds.

## 4 Discussion

Vegetable oils are added to poultry diets to increase the energy value of feed rations. Oils rich in PUFAs are more susceptible to peroxidation. Oxidised products are characterised by high ROS levels and a reduced content of fat-soluble vitamins (Panda and Cherian, 2014). Research shows that dietary supplementation with oxidised oil compromises production results (Cabel et al., 1988; Lin et al.,1989; Engberg et al., 1996; Wang et al., 1997; Anjum et al., 2004; McGill et al., 2011; Tavarez et al., 2011; Kishavy et al., 2016). In the present study, oxidised rapeseed oil as dietary stressor did not affect the growth performance of broilers, but birds whose diets were supplemented with antioxidants had higher final body weights, although the differences noted were not statistically significant. The evaluated antioxidants did not improve performance parameters. Similar results were reported by Açıkgöz et al. (2011), in whose study moderately oxidised oil did significantly affect the body weights of male broilers, feed intake and feed conversion, and a vitamin E dose of 200 mg kg${}^{-1}$ did not improve the performance of birds fed oxidised sunflower oil. Kishavy et al. (2016) demonstrated that the addition of pomegranate peel extract and sage essential oil (0.1 %) to broiler diets containing rancid oil improved performance parameters. According to Lu et al. (2014), the antioxidants ethoxyquin and propyl gallate, administered individually or in combination with vitamin E, were more effective than a vitamin E dose of 200 mg kg${}^{-1}$. In contrast, Anjum et al. (2002) and Tavarez et al. (2011) did not report an improvement in the performance of broiler chickens fed diets containing rancid soybean oil supplemented with propyl gallate and ethoxyquin.

Dietary stressors like oxidised feeds are a source of free radicals, which induce oxidative stress in birds (Lu et al., 2014). The results of numerous studies have confirmed that oxidised dietary oils decrease the concentrations of antioxidants (retinol, α-tocopherol), increase plasma MDA levels (Engberg et al., 1996) and increase the susceptibility of muscle proteins and lipids to oxidation relative to birds that are not exposed to the dietary stressor (Zhang et al., 2011; Delles et al., 2014). In the current study, dietary supplementation with vitamin E and/or polyphenols did not influence the antioxidant status or SOD activity in the blood of broiler chickens fed low-quality rapeseed oil. A vitamin E dose of 200 mg kg${}^{-1}$ increased total tocopherol content in the blood serum and breast muscles and decreased TBARS values in the liver and muscles. Broilers fed diets containing oxidised rapeseed oil, supplemented with vitamin E and polyphenols or polyphenols alone, were characterised by higher GSH-Px activity in the blood, higher tocopherol and vitamin E levels in the liver, and lower TBARS values in the breast muscles, which indicates that polyphenols effectively preserve vitamin E. According to Açıkgöz et al. (2011) and Tavarez et al. (2011), a vitamin E dose of 200 mg kg${}^{-1}$ or antioxidants (propyl gallate, ethoxyquin) did not cause differences in vitamin E levels or TBARS values in the blood or liver of birds fed rancid soybean oil. In a study by Lu et al. (2014), dietary supplementation with 200 mg kg${}^{-1}$ of vitamin E increased vitamin E concentration in breast muscles and decreased TBARS values in the blood of birds fed low-quality oil.

In the present study, polyphenols contributed to an increase in the carcass dressing percentage of broilers fed oxidised rapeseed oil, which could suggest that polyphenols exert protective effects against the harmful consequences of peroxidation products. Rancid oil, with or without antioxidants, had no effect on carcass quality. Birds fed diets with the addition of vitamin E were characterised by higher gizzard weight and higher pH of gizzard digesta. Açıkgöz et al. (2011) and Tavarez et al. (2011) did not observe differences in carcass dressing percentage, carcass weight or breast muscle weight of broilers whose diets contained rancid oil and were supplemented with vitamin E or antioxidants (propyl gallate and ethoxyquin). Zduńczyk et al. (2002) did not report differences in the carcass dressing percentage or abdominal fat content of turkeys fed oxidised oil. In the work of Anjum et al. (2004) and Racanicci et al. (2008), chickens fed oxidised or fresh oil did not differ in carcass dressing percentage or gizzard weight. Liver weight increased in birds given low-quality oil (Anjum et al., 2004). In the literature, there is a general scarcity of studies investigating the effect of polyphenols on carcass dressing percentage and the structure of the gastrointestinal tract in broilers fed low-quality dietary oils.

Peroxidation induces changes in the fatty acid profile of oils and fats. Low-quality oils with high AV and POV are sources of free radicals that exert adverse effects on unsaturated fatty acids and meat quality. Oxidative damage compromises the quality of poultry meat, including its colour, structure and water-holding capacity, and increases drip loss (Holownia et al., 2003; Sihvo et al., 2013; Zhang et al., 2011). In the current study, low-quality rapeseed oil increased pH${}_{15}$ values and the rate of change in the pH of breast muscles. After 24 h, high acidity was noted only in the muscles of broilers whose diets were supplemented with vitamin E relative to the birds receiving polyphenols. The pH${}_{15}$ values of muscles were characteristic of normal meat in groups I and III and were indicative of DFD (dark, firm and dry) meat in the remaining groups. The pH${}_{24}$ values were indicative of normal meat in all samples. The rate of change in pH${}_{24}$ values was lowest in broilers that did not receive rancid oil and was highest in the breast muscles of broilers fed diets containing rancid oil, supplemented with polyphenols and vitamin E. The breast muscles of broilers receiving polyphenols were lighter in colour, which could suggest that polyphenols inhibited the oxidation of haem pigments and prevented meat darkening. Vitamin E increased the contribution of yellowness in the breast muscles of broilers. The content of fat and crude ash was lower in the muscles of broilers that were not given oxidised oil or antioxidants relative to the remaining groups. In a study by Zhang et al. (2011), vitamin E did not affect the pH, colour or the water-holding capacity of meat in chickens fed oxidised oil. Dietary supplementation with tocopherols decreased natural drip loss in the breast muscles of birds. The muscles of broilers fed oxidised oil were characterised by higher water-holding capacity and higher natural drip. In the work of Tavarez et al. (2011), the addition of propyl gallate and ethoxyquin to feeds containing oxidised oil did not affect the pH or the water-holding capacity of meat but decreased the value of parameter $L^{*}$ (meat lightness). Lu et al. (2014) found that a combination of antioxidants (vitamin E, ethoxyquin, propyl gallate) increased the pH of breast muscles. A vitamin E dose of 200 mg kg${}^{-1}$ decreased the contribution of yellowness ($b^{*}$) and natural drip loss in the breast muscles of chickens fed low-quality oil. The analysed muscle samples did not differ in lightness or redness values (Lu et al., 2014).

In the present study, the content of PUFAs was higher and the AI was lower in chickens that did not receive oxidised oil and in broilers whose diets were supplemented with vitamin E alone or vitamin E and polyphenols relative to birds that were fed oxidised oil without antioxidants. Similar observations were made by Sheehy et al. (1994) in whose study, PUFA concentrations were higher in the lung tissue of chicks receiving fresh oil and in birds given oxidised oil with vitamin E, and they were lowest in the group of birds fed oxidised oil without antioxidants. Racanicci et al. (2008) also reported a decrease in the concentration of C18:2 PUFAs in the thigh muscles of broilers fed oxidised oil. The peroxidisability index is a measure of the susceptibility of lipids to oxidation and of the auto-oxidation of fatty acids. In our study, the PI percentage and level of PUFAs was higher in the breast muscle of the chickens fed diets without rancid oil. Similarly, Batkowska et al. (2011) and Sosnówka-Czajka et al. (2017) found the PI value to be higher in the muscle of birds with a higher PUFA content.

## 5 Conclusions

It can be concluded that the applied antioxidants had no effect on the growth performance of chickens fed oxidised oil. Increased dietary inclusion levels of vitamin E and/or polyphenols improved the antioxidant status in the blood and increased the content of non-enzymatic antioxidants in the liver and breast muscles of broilers fed low-quality oil. The tested antioxidants had no influence on carcass quality parameters in chickens fed oxidised oil. However, birds fed diets with the addition of vitamin E were characterised by higher gizzard weight and higher pH of gizzard digesta. Dietary supplementation with vitamin E and polyphenols or polyphenols alone contributed to a lighter colour and lower pH of breast muscles and an increase in the content of fat and ash in the breast muscles of broilers fed oxidised oil. The breast muscles of birds receiving supplemental vitamin E were characterised by higher concentrations of n-6 PUFAs and DFAs, a more desirable DFA/OFA ratio, and a lower atherogenicity index. Polyphenols combined with vitamin E can be a valuable component of diets for broiler chickens when the problem of low-quality oil occurs.

## Data Availability

The data sets are available upon request from the corresponding author.
